# Cryptic Hybridization Dynamics in a Three‐Way Hybrid Zone of *Dinopium* Flamebacks on a Tropical Island

**DOI:** 10.1002/ece3.70716

**Published:** 2024-12-23

**Authors:** Rashika W. Ranasinghe, Sampath S. Seneviratne, Darren Irwin

**Affiliations:** ^1^ Department of Zoology, Biodiversity Research Centre University of British Columbia Vancouver British Columbia Canada; ^2^ Department of Zoology & Environment Sciences, Faculty of Science, Avian Sciences & Conservation University of Colombo Colombo Sri Lanka

**Keywords:** Asian woodpeckers, *Dinopium*, hybridization, island biogeography, Picidae, speciation

## Abstract

Island ecosystems have emerged as vital model systems for evolutionary and speciation studies due to their unique environmental conditions and biodiversity. This study investigates the population divergence, hybridization dynamics, and evolutionary history of hybridizing golden‐backed and red‐backed *Dinopium* flameback woodpeckers on the island of Sri Lanka, providing insights into speciation processes within an island biogeographic context. Utilizing genomic analysis based on next‐generation sequencing, we revealed that the *Dinopium* hybrid zone on this island is a complex three‐way hybrid zone involving three genetically distinct populations: two cryptic populations of golden‐backed *D. benghalense* in the north and one island‐endemic red‐backed population of *D. psarodes* in the south of Sri Lanka. Our findings indicate asymmetric introgressive hybridization, where alleles from the southern *D. psarodes* introgress into the northern *D. benghalense* genome while phenotype remains adapted to their respective northern arid and southern wet habitats. The discovery of two genetically distinct but phenotypically similar *D. benghalense* populations in northern Sri Lanka highlights the process of cryptic population differentiation within island ecosystems. These populations trace their ancestry back to a common ancestor, similar to the Indian form *D. b. tehminae*, which colonized Sri Lanka from mainland India during the late Pleistocene. Subsequent divergence within the island, driven by selection, isolation by distance, and genetic drift, led to the current three populations. Our findings provide evidence of cryptic diversification and within‐island population divergence, highlighting the complexity of hybridization and speciation processes. These findings further emphasize the intricate nature of evolutionary dynamics in island ecosystems.

## Graphical abstract

This study explores population divergence and hybridization dynamics across the golden‐backed ( *Dinopium benghalense* ) and red‐backed ( *D. psarodes* ) flameback woodpecker hybrid zone in Sri Lanka. The genomic analysis uncovers three‐way hybridization among two *D. benghalense* populations in the north and one island‐endemic *D. psarodes* population in the south, demonstrating asymmetric introgressive hybridization. These findings provide insights into cryptic population differentiation and the complex evolutionary processes driving divergence within island ecosystems.

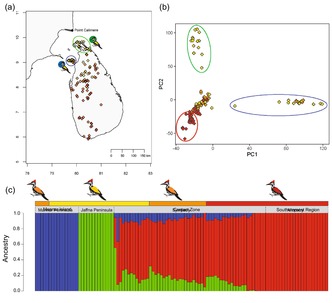

## 1 Introduction

Islands and archipelagoes have long been recognized as natural laboratories for exploring the intricate processes of speciation (Darwin 1859; Grant and Grant 2014; MacArthur and Wilson 1963; Whittaker and Fernández‐Palacios 2007). The unique features of island ecosystems, characterized by geographical isolation, limited resources, variable community structure, and a variety of distinct ecological niches compared to continents, accelerate speciation and evolution (Borregaard et al. 2017; Losos and Ricklefs 2009; Reudink et al. 2021; Stroud and Losos 2016). This distinctive combination of factors makes islands particularly well‐suited for studying key determinants shaping the pace and patterns of speciation (Aleixandre, Hernández Montoya, and Milá 2013; Coyne and Orr 2004; Coyne and Price 2000; Grant and Grant 1996; Losos and Ricklefs 2009).

Evolutionary mechanisms such as the founder effect, adaptive radiation, isolation coupled with genetic drift, and hybridization play pivotal roles in shaping biodiversity on islands (Coyne and Orr 2004; Grant and Grant 1996; Losos and Ricklefs 2009; Schluter 2000). The founder effect is an influential mechanism that occurs when a small group of individuals establishes a population on an island (Diamond 1970; Mayr 1963; Sendell‐Price et al. 2021). The founders can then expand into a bigger population while diverging from their source population. The genetic makeup of this founding population may differ significantly from the original mainland population due to the limited genetic variation of the founders and the small population size (Chaturvedi et al. 2021). The founder effect could lead to the emergence of distinctive genetic, phenotypic, physiological, and behavioral characteristics within the island population, contributing to the process of speciation through divergence (Losos and Ricklefs 2009). Island colonization can become intricate with repeated and back‐and‐forth colonization between the mainland and the island (Wickramasinghe et al. 2017). Additionally, a smaller number of colonization events from the mainland to the island over a long period allows island biotas to evolve reproductive isolation and the independent colonist groups to proliferate into multiple species (Coyne and Orr 2004; Grant 1981).

Adaptive radiation is another crucial mechanism observed among islands, wherein a single ancestral species rapidly diversifies to exploit different ecological opportunities among different island environments (Osborn 1902; Schluter 2000; Stroud and Losos 2016). Islands provide varied ecological niches and environmental conditions, which offer ample opportunities for the evolution of multiple species adapted to different niches (Stroud and Losos 2016). This phenomenon facilitates the rapid proliferation of species, each specializing in exploiting specific ecological resources. As a result, islands often host a remarkable diversity of species, each finely tuned to its unique ecological niche (Gillespie 2016; Losos and Mahler 2010; Losos and Ricklefs 2009; Schluter 2000; Stroud and Losos 2016; Yoder et al. 2010).

In island environments, several types of isolation mechanisms can work alongside genetic drift to drive speciation (Martín‐Queller et al. 2017; Sobel et al. 2010). Geographical isolation is significant, as islands are separated from mainland counterparts (Gillespie, Claridge, and Roderick 2008; Yamada and Maki 2012). Ecological isolation also plays a role, with different habitats and niches on islands leading to the evolution of distinct populations adapted to specific ecological conditions (Hendry 2004; Steinbauer et al. 2012, 2016; Steinbauer, Irl, and Beierkuhnlein 2013; Thorpe, Surget‐Groba, and Johansson 2008). Behavioral isolation arises due to unique mating behaviors or courtship rituals developed by isolated island populations (Blankers, Oh, and Shaw 2018; Uy, Irwin, and Webster 2018). Temporal isolation occurs if populations on the same island breed at different times, further reducing gene flow (Sirkiä et al. 2018). Additionally, genetic drift can amplify the effects of these isolation mechanisms by promoting random changes in allele frequencies within small island populations (Czekanski‐Moir and Rundell 2019; Hoso 2012; Losos and Ricklefs 2009; Naciri and Linder 2020).

Hybridization, interbreeding between individuals from different populations or species, is a dynamic process that can occur during colonization or subsequent interactions between divergent populations (Barton and Hewitt 1985; Grant and Grant 1996). When hybrids between the two species or populations have phenotypes that are a better fit to the environment than the parental species, hybrids might become a new species, a process known as “hybrid speciation” (Grant and Grant 1996; Howarth and Baum 2005; Ottenburghs 2018; Vallejo‐Marín and Hiscock 2016). Backcrossing of hybrids with parental populations or among hybrids can further contribute to the formation of new species by moving advantageous alleles and genes between species and populations (Abbott et al. 2013; Sætre 2013). The dynamic interplay between genetic exchange and natural selection through hybridization thus promotes speciation, contributing to the rich biodiversity often observed in island ecosystems.

In this study, we used hybridizing *Dinopium* flameback woodpeckers (Family: Picidae) on the island of Sri Lanka as a distinctive model system to examine patterns of population divergence and hybridization dynamics, shedding light on the process of speciation. Sri Lanka, historically known as Ceylon, is a continental island situated southeast of the Indian subcontinent and separated from the mainland of India by the Gulf of Mannar and the Palk Strait. Throughout the Pleistocene Era, fluctuations in sea levels caused by the cycles of Ice Age glaciations periodically connected and disconnected Sri Lanka from India (Banerjee 2000; Karanth 2003; Miller et al. 2020). These land bridge formations enabled the migration of various species between the two land masses, leading to divergent evolution (Jha et al. 2021; Lajmi et al. 2019; Meegaskumbura et al. 2019; Sholihah et al. 2021; Sudasinghe, Dahanukar, et al. 2021; Sudasinghe, Raghavan, et al. 2021; Weigelt et al. 2016; Wickramasinghe et al. 2017). This combination of geographic isolation and historical connectivity has made Sri Lanka an ideal location for studying speciation and adaptive radiation.

The island hosts two distinct species of *Dinopium* flamebacks, each exhibiting conspicuous color variations: the endemic red‐backed *D. psarodes* (Red‐backed Flameback), prevalent in the southern region, and the golden‐backed *D. benghalense* (Black‐rumped Flameback), distributed in the northern region (del Hoyo et al. 2014; Fernando, Irwin, and Seneviratne 2016; Freed et al. 2015). Notably, these two species hybridize in the north‐central region of the island where their ranges overlap, giving rise to a diverse array of plumage colors spanning from red to orange to golden‐yellow (Fernando and Seneviratne 2015; Freed et al. 2015). This phenotypic diversity within the hybrid zone, which encompasses both parental forms and a multitude of different hybrid classes, has been described as a “hybrid swarm” by Freed et al. (2015). In a comprehensive review of 148 studies employing the term “hybrid swarm,” Campbell et al. (2024) suggested that this term represents a specific form of hybridization, often used interchangeably with “hybrid zone,” as no clear distinction exists between the two. Several studies have documented the distinct phenotypic variations among flamebacks within and surrounding the hybrid zone, highlighting the presence of crimson‐red ( *D. psarodes* ) and golden‐yellow ( *D. benghalense* ) parental forms, as well as a diverse array of at least eight shades of orange plumage in hybrids (Fernando and Seneviratne 2015; Freed et al. 2015). Moreover, mitochondrial haplotype differences have been observed among parental forms, with distinct clusters identified in northern and southern regions, alongside a mixture of northern and southern haplotypes within the contact zone (Fernando, Irwin, and Seneviratne 2016).

In the present study, we employed next‐generation sequencing techniques to address several key questions. Firstly, we examined the nuclear genomic differentiation among populations of *Dinopium* flamebacks in Sri Lanka. Secondly, we explored the association between genetic variation and plumage color variations across the hybrid zone. Lastly, we elucidated the evolutionary history of the hybridizing *Dinopium* flamebacks in Sri Lanka. Our genomic analysis revealed a previously unrecognized genomic divergence among *Dinopium benghalense* populations in the north, indicating that the hybridization dynamics in Sri Lanka constitute a three‐way hybridization system involving three genetically distinct populations. Specifically, two cryptically differentiated populations of golden‐backed *D. benghalense* inhabit the north, alongside one red‐backed endemic *D. psarodes* population in southern Sri Lanka. These populations all trace their ancestry back to a common ancestor related to present‐day *D. b. tehminae*, which colonized from mainland India to Sri Lanka during the late Pleistocene.

## 2 Materials and Methods

### 2.1 Field Sampling

To investigate nuclear genomic differentiation across the *Dinopium* hybrid zone, we conducted extensive field sampling of flameback woodpeckers across Sri Lanka, spanning from northern (9.46 °N, 80.07 °E) to southern (6.04 °N, 80.38 °E) regions. A total of 141 birds were captured, comprising 46 individuals of *D. benghalense* , 62 endemic *D. psarodes* , and 33 phenotypic hybrid individuals (Figure S1a). Field sampling was conducted under the permit of the Forest Department (permit no: R&E/RES/NFSRC/2015/04) and the Department of Wildlife Conservation (permit no: WL/3/2/19/13) in Sri Lanka. All birds were captured using mist nets. Locally recorded flameback vocalization playbacks and life‐size wooden decoys were used to attract the birds to the mist nets. From each bird, we collected approximately 50 μL of blood from the brachial vein, preserving it in either Queen's lysis buffer (0.01 M Tris, 0.01 M NaCl, 0.01 M EDTA, 1% n‐lauroylsarcosine, pH 7.5) or on autoclaved Whatman filter paper (catalog number 1001 125). Blood on filter paper was air‐dried and then stored securely in opaque envelopes.

### 2.2 DNA Extraction and Sequencing

Blood samples were processed at the Laboratory for Molecular Ecology and Evolution, University of Colombo, prior to DNA being sent to the Biodiversity Research Centre, University of British Columbia (export permit WL/3/2/44/18). DNA extraction was performed using a standard phenol‐chloroform extraction. For dried blood on filter paper, we began by finely chopping a single drop of the blood patch (approximately 10 mm in diameter) and dissolving the fragments in 250 μL of Queen's lysis buffer, followed by the standard phenol‐chloroform extraction protocol. For the sequencing of nuclear genomes, we employed the Genotyping‐by‐Sequencing (GBS) technique as outlined by Elshire et al. (2011) and Alcaide et al. (2014). In brief, GBS library preparation involved the digestion of genomic DNA and ligation of barcodes and adaptors, achieved by mixing 6 μL of adaptors, 6 μL of barcodes, 1 μL of Pst I restriction enzyme, 2 μL of 10× buffer, and 5 μL of 20 ng/μL gDNA template per sample. This mixture was incubated at 37°C for 2 h. Digested DNA fragments were ligated with adaptors and barcodes using T4 ligase, with incubation at 22°C for 1 h, followed by incubation at 65°C for 10 min. The resulting reaction mixture was then cleaned using AMPure XP beads (Beckman‐Coulter). For PCR amplification, we combined 5 μL of 5× Phusion Buffer, 0.5 μL of 10 mM dNTPs, 1.25 μL of forward and reverse primers, 12.75 μL of UltraPure water, and 0.25 μL of PhusionTaq per sample. Amplification occurred under this thermal protocol: initial denaturation at 98°C for 30 s, followed by 18 cycles of denaturation at 98°C for 10 s, annealing at 65°C for 30 s, and extension at 72°C for 5 min, followed by holding at 4°C. We pooled all the prepared samples and subjected them to electrophoresis on a 2% agarose gel to isolate DNA fragments ranging from 400 to 500 base pairs. Afterward, we extracted DNA fragments from the gel using the Qiagen Gel Extraction Kit. GBS sequencing was performed at two sequencing facilities. A subset of the samples was sequenced at Genome Quebec, Canada, while the remaining samples were sequenced at The Centre for Applied Genomics (TCAG), Ontario, Canada. In both facilities, we conducted paired‐end sequencing with 150‐base pair reads using the Illumina Hiseq4000 at Genome Quebec and the Illumina NovaSeqSP6000 at TCAG.

### 2.3 SNP Genotyping

We conducted an in‐depth analysis of the resultant GBS reads using custom scripts available at https://doi.org/10.5061/dryad.4j2662g, a resource provided by Irwin et al. (2018). In brief, we initiated the analysis by demultiplexing the raw GBS reads using a custom Perl script. Subsequently, we performed read trimming with Trimmomatic 0.32 (Bolger, Lohse, and Usadel 2014), employing specific parameters: TRAILING:3, SLIDINGWINDOW:4:10, and MINLEN:30. We then used BWA‐MEM with default settings to align the reads to the Downy woodpecker ( *Picoides pubescens* ) reference genome (NCBI Assembly: https://www.ncbi.nlm.nih.gov/assembly/GCA_014839835.1). Identification of single nucleotide polymorphisms (SNPs) and genotype calling was achieved through HaplotypeCaller and GenotypeGVCFs commands of GATK 3.8 (McKenna et al. 2010). We conducted subsequent filtering steps using VCFtools and GATK (utilizing the VariantFiltration command). This multi‐step filtering process involved the following criteria: removal of indels, selection of only bi‐allelic loci, exclusion of questionable loci (QD < 2; or MQ < 20), elimination of sites where more than 60% of individuals had missing genotypes, removal of individuals with more than 30% missing genotypes, and keeping SNPs with a minor allele count (MAC) of at least 2.

### 2.4 Analysis of Genomic Data

#### 2.4.1 Examination of Genomic Differentiation Between Populations

We performed a Principal Component Analysis (PCA) to elucidate the nuclear genomic relationships within and across the *Dinopium* hybrid zone using R scripts described by Irwin et al. (2018), implemented in the R programming language (R Core Team 2022). To examine the genomic ancestry of each individual, we calculated the admixture proportions using ADMIXTURE 1.3.0 (Alexander and Lange 2011). Initially, the dataset underwent linkage disequilibrium pruning, removing SNPs with a correlation coefficient (*R* ^2^) exceeding 0.1 with any other SNP within a 50‐SNP sliding window, advancing by 10 SNPs at each iteration. Then admixture analysis was performed with cross‐validation across *K* values ranging from 1 to 5 to determine the optimal number of ancestral populations. The standard error of the cross‐validation (CV‐error) estimate was computed for each *K* value, and the *K* value with the lowest CV‐error was determined as the optimal estimate for the number of ancestral populations in the dataset. In our analysis, *K* = 3 emerged as the optimal estimate for the number of ancestral populations.

To estimate the degree of genetic differentiation both between and within populations, we calculated (1) relative population differentiation (*F*_ST_), (2) absolute nucleotide distance between populations (*D*_*xy*_), and (3) within‐population nucleotide variation (*π*). For these calculations, we included southern *D. psarodes* , *D. benghalense* from the Jaffna peninsula, and *D. benghalense* from Mannar island, each with respective ancestry proportions > 0.99998 in the ADMIXTURE analysis. Sequencing data were evaluated using non‐overlapping sliding windows, each encompassing 10,000 base pairs, following the methodology detailed in Irwin et al. (2018).

#### 2.4.2 Cline Analysis

We elucidated the transition of *D. psarodes* ancestry and phenotype across the hybrid zone using the R package HZAR, following the methodology outlined by Derryberry et al. (2014). HZAR utilizes the Metropolis–Hastings Markov chain Monte Carlo (MCMC) algorithm to fit genomic or morphological data to a suite of equilibrium cline models. We used *D. psarodes* ancestry proportions obtained from ADMIXTURE analysis (*K* = 3) to produce the genomic cline. To establish the phenotypic cline, we used photographs of each bird to quantify luminosity (*L*) ranging from black (0) to white (100), a color matrix (*a*) spanning from green (negative) to red (positive), and a second color matrix (*b*) ranging from blue (negative) to yellow (positive). This was achieved by utilizing the LAB color space in Adobe Photoshop CC (2022), following the approach detailed by Wang et al. (2020). A Principal Component Analysis (PCA) was conducted on the collected *L*, *a*, and *b* values. The principal component (PC) that explained the most phenotypic variation from red to golden‐yellow (PC1 in this study) was considered as the representative phenotype (Figure S2a). For both genomic and phenotypic clines, we computed geographic distances from Point Calimere (10.2864° N, 79.8299° E) in south India to each sampling location using the ruler tool in Google Earth Pro 7.3.6. Maximum likelihood estimates were obtained for cline centers and cline widths, and Akaike Information Criteria (AIC; Akaike 1974) were computed for each cline fit to identify the best‐fitting models.

#### 2.4.3 Estimation of Divergence Times

We investigated the evolutionary history of *Dinopium* flamebacks in Sri Lanka, encompassing population divergence between mainland India and Sri Lanka, by estimating population divergence times among *Dinopium benghalense* subspecies in India (*D. b. dilutum*, *D. b. puncticolle*, *D. b. tehminae*, and *D. b. benghalense*) and the three populations of *Dinopium* flamebacks in Sri Lanka. A phylogenetic tree was created based on genomic variant data (SNPs) using BEAST2 2.7.5 (Bouckaert et al. 2019) with the add‐on package SNAPP (Bryant et al. 2012). Genotype data for *D. benghalense* subspecies in India were obtained from tissue samples (toe pad samples) collected from various institutions, including the Field Museum of Natural History (three individuals of *D. b. benghalense*, and two individuals of *D. b. puncticolle*), Harvard Museum of Comparative Zoology (two individuals of *D. b. dilutum*), and the Smithsonian National Museum of Natural History (one individual each of *D. b. puncticolle* and *D. b. dilutum*, and two individuals of *D. b. tehminae*). We prepared the toe pad samples for standard phenol‐chloroform DNA extraction by finely chopping and incubating with 250 μL of Queen's lysis buffer, 15 μL of NaCl, and 30 μL of proteinase K at 55°C for approximately 24 h, with periodic vortexing. Subsequent DNA extraction, GBS library preparation, sequencing, and SNP genotyping were performed as previously described. Three individuals were chosen from each of the three *Dinopium* populations in Sri Lanka based on their ancestry proportion, which was ≥ 0.99998 in the ADMIXTURE analysis. Additionally, three individuals of *Chrysocolaptes stricklandi* (Crimson‐backed Flameback) were included as the outgroup. The SNP genotype file (VCF file) containing all these individuals was filtered using VCFTOOLS to retain only bi‐allelic sites. Missing data were imputed using Beagle‐5.4 (Browning et al. 2021). A ruby script (snapp_prep.rb script), as described in Stange et al. (2018), was employed to prepare SNAPP input files with a run chain length of 500,000 MCMC iterations. This script facilitated the time calibration of the species tree estimated by SNAPP through the application of age constraints on one or more divergence events. To specify age constraints for divergence events within *Dinopium* populations, we employed ‘crown’ divergence with a normal distribution. In the constraint file, we delineated the estimated time to the most recent common ancestor between the genus *Dinopium* and the genus *Chrysocolaptes* as approximately 12 ± 1.5 Myr, as estimated by Fuchs et al. (2007). Subsequently, TreeAnnotator–2.7.5 (Drummond and Rambaut 2007) was utilized to generate a maximum‐clade‐credibility tree, and the final tree was visualized in R using the ggtree package (Yu et al. 2017).

#### 2.4.4 Calculation of Pairwise Nei's Genetic Distances

We further explored the historical relationships among *D. benghalense* in India and *Dinopium* populations in Sri Lanka by calculating pairwise Nei's genetic distances using the *adegenet* −2.0.0 R package (Jombart and Collins 2015). For this analysis, we included the same *D. benghalense* samples from India as described above in the phylogenetic analysis. From the Sri Lankan populations, we included only allopatric individuals with ancestry proportion ≥ 0.99998, comprising 37 samples of southern endemic *D. psarodes* , 13 samples of *D. benghalense* in Mannar, and 16 samples of *D. benghalense* in Jaffna. Nei's genetic distances were calculated based on 257,895 SNP sites across all these samples. The adegenet package computes pairwise Nei's genetic distances by conducting a Mantel test to assess isolation‐by‐distance (IBD) between a matrix of genetic distances, which are determined based on gene identity between populations (Jombart and Collins 2015; Nei 1972, 1978).

## 3 Results

**FIGURE 1:**
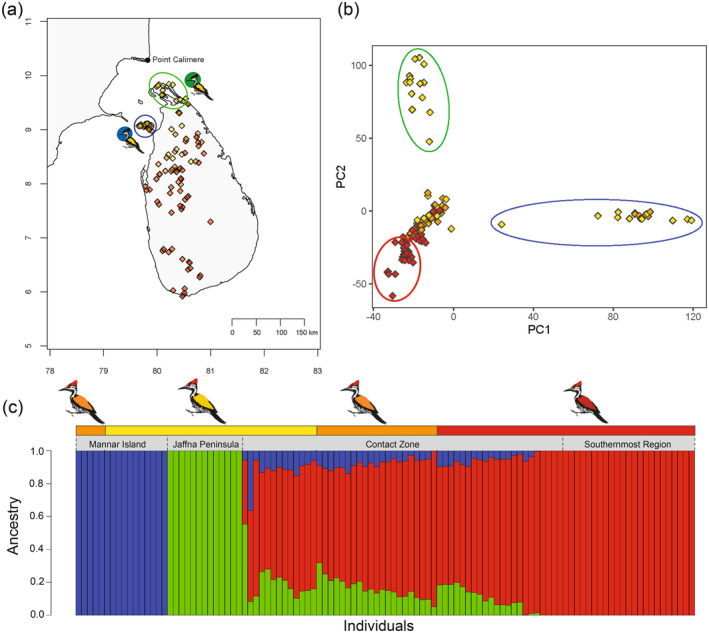
(a) Sampling map showing capture locations of *Dinopium* flamebacks included in the downstream analysis after filtering for missing genotype data (*n* = 108). Two circles highlight the geographic regions with genetically distinct northern *Dinopium* populations; Mannar Island (blue circle) and Jaffna peninsula (green circle). Please note that Supporting Information include maps of all the sampled individuals (*n* = 141; Figure S1a) and those excluded from the analysis (*n* = 33; Figure S1b). (b) Principal component analysis (PCA) based on 734,806 SNPs illustrating genetic differentiation among flamebacks across the island. Birds captured from the Jaffna peninsula are enclosed within a green ellipse, while those from the Mannar Island are in a blue ellipse. A red ellipse encircles the allopatric red‐backed *D. psarodes* , which inhabits the southernmost region of the island. In both the sampling map and PCA, colors represent the phenotype of the bird; red for red‐backed (*n* = 45), yellow for golden‐backed (*n* = 37) and orange for orange‐backed (*n* = 26). (c) Ancestry proportions inferred from ADMIXTURE analysis (*K* = 3). Red represents *D. psarodes* ancestry, green indicates *D. benghalense* ancestry from Jaffna, and blue represents *D. benghalense* ancestry from Mannar. The top‐colored bar indicates the plumage color of each bird (red for red‐backed, yellow for golden‐backed, and orange for orange‐backed), while the gray bar below it shows the capture location: Mannar island, Jaffna peninsula, contact zone, or southernmost regions.

The genotype‐by‐sequencing analysis conducted on 141 *Dinopium* flameback individuals yielded a total of 1,251,423 SNPs for 108 individuals after filtering out of 33 individuals due to higher proportion of missing data. The sampling locations of individuals included in the analysis are shown in Figure 1a, while the locations of excluded individuals are provided in Figure S1b. Further filtering to retain only those SNPs with a maximum of 10% missing data resulted in a final dataset of 734,806 SNPs. SNP‐based PCA revealed the presence of three genetically distinct clusters of *Dinopium* flamebacks in Sri Lanka (Figure 1b). Two of these clusters corresponded to flamebacks from the northern regions of the island, each representing separate geographic populations: one from Mannar island on the northwestern coast (9.2° N, 79.5° E) and the other from the Jaffna peninsula in the far north (9.3° N, 80.1° E). The third cluster comprised all remaining sampled individuals from the island, including the southern endemic red‐backed *D. psarodes* as well as red, yellow, and orange‐backed flamebacks sampled from the contact zone. Notably, within this cluster, a clear separation was observed between the allopatric *D. psarodes* (which inhabit the southernmost part of the island) and individuals from the contact zone (Figure 1b). Principal Component (PC) 1 distinguished the flameback population on Mannar island from other populations, accounting for 2.7% of the variance observed among the samples. PC 2 accounted for 2.1% of the variance, separating the population on Jaffna peninsula from the remaining samples. All the birds captured from the contact zone were positioned on the PCA plot closer to the red‐backed group but in the direction of the two golden‐backed groups, reflecting intermediate genetic affiliations (Figure 1b).

**FIGURE 2:**
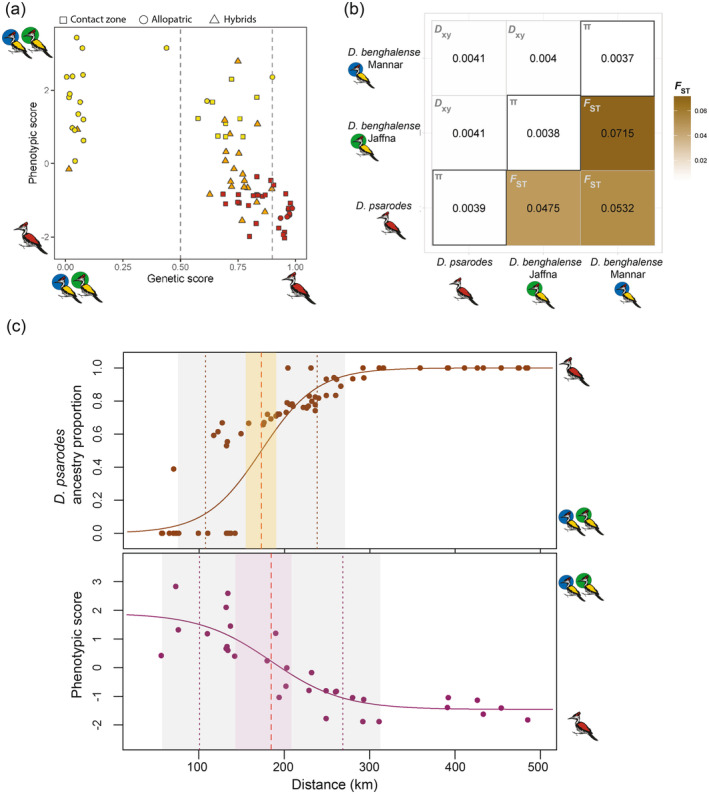
(a) Scatter plot illustrating the relationship between phenotypic and genetic scores of *Dinopium* individuals across the hybrid zone. Phenotype of the bird is represented by colors: Red; red‐backed birds, yellow; golden‐backed birds, and orange; orange‐backed birds. Shapes indicate whether birds are allopatric (circle), contact zone (square), or hybrid (triangle). (b) Heatmap illustrating *F*_ST_, *D*_*xy*_, and *π* values for *Dinopium* Flameback populations. The diagonal squares in the center display *π* values for each population. Squares located in the top left corner depict *D*_*xy*_ between each population pair. Squares positioned in the bottom right corner depict *F*_ST_ between each population pair. The color gradient of the *F*_ST_ values represents the magnitude, with darker colors indicating higher *F*_ST_ values. (c) Genomic and phenotypic clines. The top panel illustrates the genomic cline, while the bottom panel depicts the phenotypic cline. In both panels, the red dashed line represents the center of the cline, while the black dotted lines on either side indicate the boundaries of the cline width. The colored regions denote the likelihood of the cline center (khaki rectangle for the genomic cline, maroon rectangle for the phenotypic cline), while the gray regions represent the likelihood of the cline width boundaries. In the top graph, each dot represents an individual bird, whereas in the lower graph, each dot represents the average phenotype score for each locality.

Admixture analysis further corroborated these findings, identifying three distinct genomic ancestries that are concordant with the genetic clusters observed in the PCA (Figure 1b—shown in ellipses in the PCA; two northern *D. benghalense* populations [green and blue ellipses] and the southernmost *D. psarodes* [red ellipses]) (Figure 1c). It also indicated that birds in the contact zone were the result of extensive admixture among the three populations. Notably, only one individual exhibited > 50% *D. benghalense* ancestry, while all the remaining admixed individuals showed > 50% *D. psarodes* ancestry. This finding implies that most of the admixed individuals are multigenerational backcrosses to the red‐backed *D. psarodes* (Figure 1c). Additionally, the analysis identified orange‐backed individuals captured from Mannar Island as having nearly pure *D. benghalense* Mannar ancestry, with ancestry proportion > 0.99998 (Figure 1c). Moreover, all phenotypically golden‐backed and orange‐backed flamebacks from the contact zone had an average of > 70% red‐backed *D. psarodes* ancestry (Figure 2a). These findings reveal discrepancies between phenotype and genotype across both allopatric populations and birds in the contact zone (Figure 2a).

The cline analysis revealed that both genomic and phenotypic clines exhibited broad widths. The genomic cline was estimated to be 130.75 km wide, ranging from Kilinochchi (107.51 km: 9.3803° N, 80.3770° E) to Eppawala (238.26 km: 8.1441° N, 80.4119° E), while the plumage cline spanned approximately 167.64 km, from Nallur (100.93 km: 9.4503° N, 80.2781° E) to Melsiripura (268.56 km: 7.6450° N, 80.5077° E) (Figure 2c). Consequently, the genomic cline width was approximately 37 km narrower than the phenotypic cline width. The estimated cline centers for both genomic and phenotypic data were located in the Vavunia District (8.7542° N, 80.4982° E) in northern Sri Lanka. While the point estimate of the genomic cline center was shifted approximately 12 km north of the phenotypic cline center, this difference is not statistically significant (genotypic cline center: 172.88 km [CI of estimate: 153.86–191.07 km]; phenotypic cline center: 184.74 km [CI of estimate: 142.18–209.22 km]).

All three genetic clusters displayed a similar average genomic diversity within and between populations (*π* and *D*_*xy*_), suggesting recent divergence of these populations and/or substantial gene flow between them (Figure 2b). Average *F*_ST_ values indicated overall low levels of genomic differentiation but were significantly different between populations (ANOVA: *F*_2,13,194_ = 831.69, *p* < 2.2 × 10^−16^). Notably, the highest divergence was observed between the two *D. benghalense* populations in northern Sri Lanka (Mannar and Jaffna populations) with an *F*_ST_ of 0.0715 ± 0.0336. The second‐highest *F*_ST_ value was between *D. benghalense* in Mannar and the island‐endemic *D. psarodes* (*F*_ST_ = 0.0532 ± 0.0274). These findings collectively highlight the substantial genomic differentiation of *D. benghalense* in Mannar from the other two flameback populations on the island.

**FIGURE 3:**
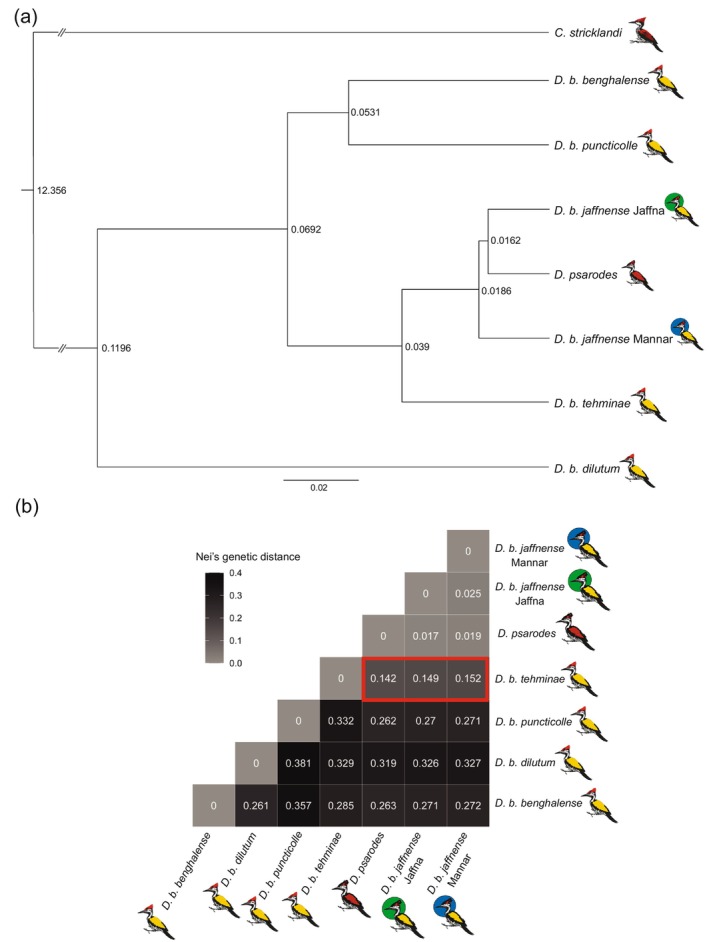
(a) Maximum‐clade‐credibility tree generated using SNAPP. Nodes on the tree represent the estimated divergence time at each node in millions of years. (b) Heatmap showing pairwise Nei's genetic distances. Color gradient represents the magnitude of the distance; darker colors signify greater genetic divergence between the two populations.

The analysis of the evolutionary history of *Dinopium* flamebacks in Sri Lanka indicated that the *Dinopium* flamebacks in Sri Lanka diverged from *D. b. tehminae* in southwestern India (Figure 3a). Consistent with these findings, Nei's genetic distance calculations also revealed similar patterns, with all three flameback populations in Sri Lanka exhibiting the lowest genetic distances to *D. b. tehminae* in India (Figure 3b). Divergence time estimations indicated that *Dinopium* in Sri Lanka likely diverged from *D. b. tehminae* approximately 39,000 years ago during the Pleistocene era (2.58 million years ago—11,700 years ago) (Figure 3a). Furthermore, the Mannar population of *D. benghalense* diverged from the other two populations in the island (namely *D. benghalense* in Jaffna and southern *D. psarodes* ) approximately 18,600 years ago. The separation between *D. benghalense* in Jaffna and southern *D. psarodes* occurred around 2400 years later, approximately 16,200 years ago (Figure 3a).

## 4 Discussion

Our findings unveiled a previously unrecognized complexity in the hybridization dynamics of *Dinopium* flamebacks on the island of Sri Lanka, challenging the conventional understanding of two‐way hybridization. Contrary to expectations, our data revealed three‐way hybridization involving the southern island‐endemic red‐backed *D. psarodes* and two genetically distinct populations of golden‐backed *D. benghalense* in northern Sri Lanka. The examination of nuclear genomic differentiation across the *Dinopium* hybrid zone sheds light on the unexpected genetic divergence in northern *D. benghalense* inhabiting the Jaffna peninsula and Mannar island. Consequently, the *Dinopium* flameback hybrid zone is revealed as a three‐way hybrid zone involving three distinct populations from two species, representing the first documented instance of such complexity in tropical *Dinopium* woodpeckers. While this phenomenon is noteworthy, it is not unprecedented among woodpeckers (Ottenburghs 2018). For instance, three‐way hybridization has been observed between red‐breasted ( *Sphyrapicus ruber* ), red‐naped ( *S. nuchalis* ), and yellow‐bellied sapsuckers ( *S. varius* ) in western Canada (Grossen et al. 2016; Natola, Seneviratne, and Irwin 2022; Seneviratne et al. 2016). However, unlike the *Dinopium* flamebacks, this sapsucker tri‐hybrid zone does not exhibit extensive hybridization. Instead, it shows evidence of selection against hybrids, preserving species boundaries among the three species (Natola, Seneviratne, and Irwin 2022). It is noteworthy that such intricate three‐species hybridization phenomena are not as rare in nature as previously assumed (Ottenburghs 2018). Instances of three‐species hybridization have been documented across various organisms, including amphibians such as European cave salamanders (genus *Speleomantes*; Bruni et al. 2023), arthropods such as ticks (*Dermacentor*; Oliver, Wilkinson, and Kohls 1972), fishes such as three‐spined sticklebacks (*Gasterosteus*; Dean et al. 2024), plants such as spruce (*Picea*; Hamilton, De La Torre, and Aitken 2015), pines (*Pinus*; Peloquin and Gartner 1984), azalea flowers (*Rhododendron*; Ureshino, Miyajima, and Akabane 1998; Zheng et al. 2021), Indian paintbrush (*Castilleja*; Hersch and Roy 2007), and other avian groups like finches (*Geospiza*; Grant and Grant 2020), warblers (*Acrocephalus*; Reifová et al. 2016), and plovers (*Charadrius*; Thies et al. 2018). These examples underscore the complexity of hybridization processes in diverse taxonomic groups.

The prevalence of backcrossing in the *Dinopium* hybrid zone is notably biased toward island‐endemic red‐backed *D. psarodes* , with even some golden‐backed flamebacks in the contact zone exhibiting > 70% *D. psarodes* ancestry. This may suggest potential survival and reproductive advantages conferred by *D. psarodes* alleles within the local environment and habitat of the contact zone. Supporting our findings, Fernando (2020) also suggested a mate choice biased toward red‐backed phenotype in the contact zone, based on a study of over 300 mated pairs. The little evidence for backcrossing to *D. benghalense* in our data may suggest potential barriers to successful reproduction or reduced hybrid fitness when backcrossing occurs with the *D. benghalense* parental species. These barriers could encompass genetic incompatibilities or ecological preferences that limit the occurrence of backcrossing to *D. benghalense* . Additionally, the observed 12 km northward shift in the genomic cline compared to the phenotypic cline likely arises from the absence of backcrosses to *D. benghalense* and implies the introgression of *D. psarodes* alleles into northern *D. benghalense* populations.

Asymmetric introgressive hybridization has been observed in other avian tri‐hybrid zones as well. For instance, the tri‐hybrid zone of Darwin's finches in the Galapagos Islands showed gene flow from *G. fortis* into *G. scandens* (Grant and Grant 2020). Additionally, in three hybridizing *Acrocephalus* species, unidirectional gene flow has been observed from Common reed warbler (*A. scripaceus*) to Marsh warbler ( *A. palustris* ) and also from *A. palustris* to Blyth's reed warbler ( *A. dumetorum* ) (Thies et al. 2018). Similarly, the *Dinopium* tri‐hybrid zone also demonstrates asymmetric introgression, where the southern *D. psarodes* genome introgresses into the northern *D. benghalense* population. This is evidenced by the presence of a predominantly southern nuclear genome combined with a northern phenotype in the contact zone. This phenomenon suggests that while genetic material may flow asymmetrically between species, there are barriers preventing the transfer of observable phenotypic characteristics.

We hypothesize that phenotype plays a significant role in adaptation of flamebacks to their habitats, aligning with Gloger's rule, which suggests that organisms inhabiting warmer, more humid environments tend to exhibit darker pigmentation, whereas those in drier, less humid areas often display lighter pigmentation (Delhey 2019). Correspondingly, we observed a darker crimson red‐backed phenotype adapted to the lowland tropical wet, high‐humidity habitats (some parts of this area fall under the per humid zone where year‐round relative humidity stayed at 100%) in the southern region, while a brighter golden‐yellow‐backed phenotype was confined the drier, arid habitats in the northern part of the island. This environmental adaptation likely drives a strong selective pressure against the northward spread of the red‐backed plumage, despite the exchange of nuclear genetic material, indicating the specific adaptation of red coloration to the wet zones of the southern island. Consequently, plumage color expression in *Dinopium* lineages might be influenced by a combination of genetic and environmental factors. Additionally, social interactions between *Dinopium* and *Chrysocolaptes* flamebacks, such as mimicry, may also influence plumage color evolution within *Dinopium* lineages (Miller et al. 2019; Prum 2014). However, both the red‐backed *C. stricklandi* (Crimson‐backed Flameback) and yellow‐backed *C. festivus* (White‐naped Woodpecker) forms of *Chrysocolaptes* are distributed across the entire island. There is no evidence of a similar distribution pattern as observed in *Dinopium*, where the yellow‐backed form is restricted to the north and the red‐backed form to the south. Future research efforts are crucial for further exploring mimicry‐driven phenotypic dynamics in these lineages.

The overall low levels of *F*_ST_, *D*_*xy*_, and *π*, as well as the similar levels of *D*_*xy*_ and *π*, suggest that the three *Dinopium* populations may have recently evolved. This evolution likely involved a combination of genetic differentiation, primarily due to geographic isolation, along with ongoing gene flow and shared ancestry. These populations may have diverged from a common ancestor, possibly due to geographic barriers and/or founder events, but have since maintained connectivity to some extent, leading to moderate genetic differentiation in the nuclear genome. The comparable levels of genetic diversity suggest that despite some divergence, the populations have retained adaptive potential and may continue to evolve in response to local selective pressures. The low level of genetic diversity, on the other hand, could be due to the result of increased rates of genetic drift and inbreeding due to lower effective population size on island populations (Frankham 1997). Similar to our findings, a low level of genetic diversity has been reported in different populations of trumpeter finch ( *Bucanetes githagineus* ) (Barrientos et al. 2014), blue tit subspecies (*Cyanistes* spp.) on the Canary Islands (Hansson et al. 2014; Stervander et al. 2015), and Cooper and Uy (2017) reported lower nuclear diversity and *F*_ST_ among recently diverged populations of *Monarcha castaneiventris* flycatchers on Solomon Islands. Additionally, the observed *F*_ST_ between each *Dinopium* population pair provides insights into the connectivity patterns between the three populations. Of the three population pairs, the higher *F*_ST_ between the two populations of *D. benghalense* suggests limited gene flow between these populations, which is consistent with the geographical isolation of Mannar island and Jaffna peninsula from each other. The lower *F*_ST_ value between *D. benghalense* in Jaffna and *D. psarodes* in the main island compared to the *F*_ST_ between *D. benghalense* in Mannar and *D. psarodes* highlights differences in the degree of geographic isolation experienced by each landmass (Mannar and Jaffna) with the main island. This disparity likely reflects variations in geographic connectivity, with Jaffna peninsula being more connected to the main island through a broader land bridge compared to the narrower connection between the Mannar Island and the main island, which is characterized by a narrow lagoon stretch.

The unexpected nuclear genomic differentiation observed in the phenotypically similar northern golden‐backed *D. benghalense* provides suggestive evidence of incipient cryptic speciation within this species complex. Cryptic speciation, characterized by subtle phenotypic differences masking significant genetic divergence, challenges traditional taxonomic methods reliant on morphology alone for species identification (Bickford et al. 2007; Fišer, Robinson, and Malard 2018; Skoracka et al. 2015). In *Dinopium*, in which distinctions between red‐backed and golden‐backed forms may be obvious to human observers, subtle variations within golden‐backed forms might elude detection based on phenotype alone. This again highlights the limitations of relying solely on visual cues for species delimitation and underscores the importance of molecular techniques in uncovering hidden biodiversity (Pulido‐Santacruz, Aleixo, and Weir 2018). Consistent with our findings, literature provides numerous empirical examples for cryptic speciation in avian groups, such as divergence of oriental greenfinch ( *Chloris sinica* ) on Oceanic islands (Saitoh et al. 2020), winter wrens ( *Troglodytes troglodytes* ) in North America (Toews and Irwin 2008), North American Bicknell's Thrush ( *Catharus bicknelli* ) and Gray‐cheeked Thrush ( *Catharus minimus* ) (Termignoni‐Garcia et al. 2022), band‐rumped storm‐petrels (*Hydrobates* spp.) (Taylor et al. 2019), and substantial genomic diversification of understory cryptic Amazonian taxa: woodcreepers (*Xiphorhynchus*), antbirds (*Willisornis*) (Pulido‐Santacruz, Aleixo, and Weir 2018), antshrikes (*Thamnophilus*) (Thom and Aleixo 2015) and hummingbirds (*Phaethornis*) (Araújo‐Silva et al. 2017). These findings emphasize that biodiversity may be more complex and nuanced than what is discernible through superficial observations.

We hypothesized that the cryptic population diversification in *D. benghalese* populations could be facilitated by either a combination of isolation‐by‐distance and genetic drift or a recent colonization event involving a sister‐species of *D. benghalense* from mainland India to the Mannar island. However, phylogenetic analysis provided evidence of shared ancestry among the three flameback populations within the island, suggesting a single historical colonization event approximately 39,000 years ago in the Pleistocene era gave rise to the *Dinopium* flamebacks in Sri Lanka. Notably, the source population for this colonization event appears to be *D. b. tehminae* (Figure 3), currently confined to the Western Ghats in southwestern India. Ripley (1949) proposed a similar scenario, suggesting that *D. benghalense* in northern Sri Lanka resulted from the colonization of *D. b. tehminae* from India and that *D. b. jaffnense* in northern Sri Lanka exhibits a phenotypic similarity to *D. b. tehminae*. However, Ripley (1949) has suggested multiple colonization events, with the first colonization event giving rise to *D. psarodes* , and the second involving *D. b. tehminae*, which contributed to the current *D. benghalense* populations in northern Sri Lanka. Our findings may reflect the signature of this second colonization event. Moreover, it is also crucial to consider the potential role of gene flow between the two species within Sri Lanka. Such gene flow could have obscured genomic differences between *D. psarodes* and *D. benghalense* , making the shared ancestry in our phylogenetic analysis possibly a result of this genetic exchange. Ripley (1949) also mentioned the possibility of recent gene flow from *D. b. puncticolle* (a third colonization event), currently inhabiting southern India, to Sri Lanka. However, our genomic analysis did not uncover any evidence supporting this colonization event. Nei's genetic distances further corroborated our phylogenetic analysis, demonstrating the closest genetic affinity between the flamebacks in Sri Lanka and *D. b. tehminae* in India compared to all other *D. benghalense* subspecies. Among the three populations in Sri Lanka, the *D. benghalense* population in Mannar exhibited the highest genetic distance from all lineages of *D. benghalense* subspecies in India. This finding suggests that the origin of *D. benghalense* Mannar birds from a recent colonization event from India is highly improbable. If such colonization had taken place, we would anticipate a lower Nei's genetic distance between the Mannar population and the invaded source population of *D. benghalense* subspecies in India. These findings suggest that the cryptic population diversification in *D. benghalense* populations is likely driven by a combination of isolation‐by‐distance and genetic drift particularly due to small population sizes.

Consequently, our findings suggest that *Dinopium* flamebacks in the island of Sri Lanka likely originated from a historical colonization event of *D. b. tehminae* from mainland India. Subsequent within‐island divergence facilitated the current distinct populations due to isolation‐by‐distance, genetic drift, and adaptation. Divergence time estimates indicated that these migration events and population divergences occurred during the late Pleistocene era (126,000–11,700 years ago), facilitated by a land bridge exposed between Sri Lanka and south India due to falling global sea levels (Banerjee 2000; Miller et al. 2020; Reuter, Harzhauser, and Piller 2021; Voris 2000). However, it's important to note that estimated divergence times may be underestimated due to ongoing gene flow between populations. Evidence of within‐island (in situ) speciation on islands has been very rare in birds (Bird et al. 2012; Coyne and Price 2000; Kisel and Barraclough 2010). For example, Coyne and Price (2000) investigated 46 islands and small archipelagoes and found little evidence of within‐island speciation in birds. However, there are a few reported historical cases, such as Emerson (2008) and Mayr (1942). In addition, Gabrielli et al. (2020) documented another in situ island speciation of gray white‐eyes ( *Zosterops borbonicus* ) on volcanic island of Reunion. Furthermore, Weerakkody et al. (2023) have reported within‐island allopatric speciation in drongo (genus: *Dicrurus*) in the island of Sri Lanka. Adding more to these examples, our study also provides an example of within‐island population divergence despite gene flow.

## Author Contributions

**Rashika W. Ranasinghe:** conceptualization (equal), data curation (lead), formal analysis (lead), methodology (equal), visualization (lead), writing – original draft (lead). **Sampath S. Seneviratne:** conceptualization (equal), methodology (equal), project administration (equal), resources (supporting), writing – review and editing (equal). **Darren Irwin:** conceptualization (equal), formal analysis (supporting), funding acquisition (lead), methodology (equal), project administration (equal), resources (lead), supervision (lead), writing – review and editing (equal).

## Conflicts of Interest

The authors declare no conflicts of interest.

## Supporting information


Data S1.

## Data Availability

All the data processing and analyzing scripts, and metadata, are available in the Dryad repository (https://doi.org/10.5061/dryad.6hdr7sr92) and the GitHub repository (https://github.com/rashikaranasinghe/GBS_data_processing_and_analysis). The raw GBS sequencing reads have been deposited in the NCBI Sequence Read Archive under BioProject accession PRJNA1139147.
