# Supplementary material for: Cryptic Hybridization Dynamics in a Three‐Way Hybrid Zone of Dinopium Flamebacks on a Tropical Island
Source: Ecol Evol. 2024 Dec 23;14(12):e70716. doi: 10.1002/ece3.70716 (PMC11664123; doi:10.1002/ece3.70716)
Supplement: Supplementary file 1 — Data S1. [file ECE3-14-e70716-s001.zip › 5 Three-way-hybridization of Dinopium flamebacks manuscript 5thJuly - Supplementary materials_without Track Changes.pdf]

Title: Cryptic Hybridization Dynamics in a Three-Way Hybrid Zone of *Dinopium* Flamebacks on a Tropical Island.

Supplementary Materials :

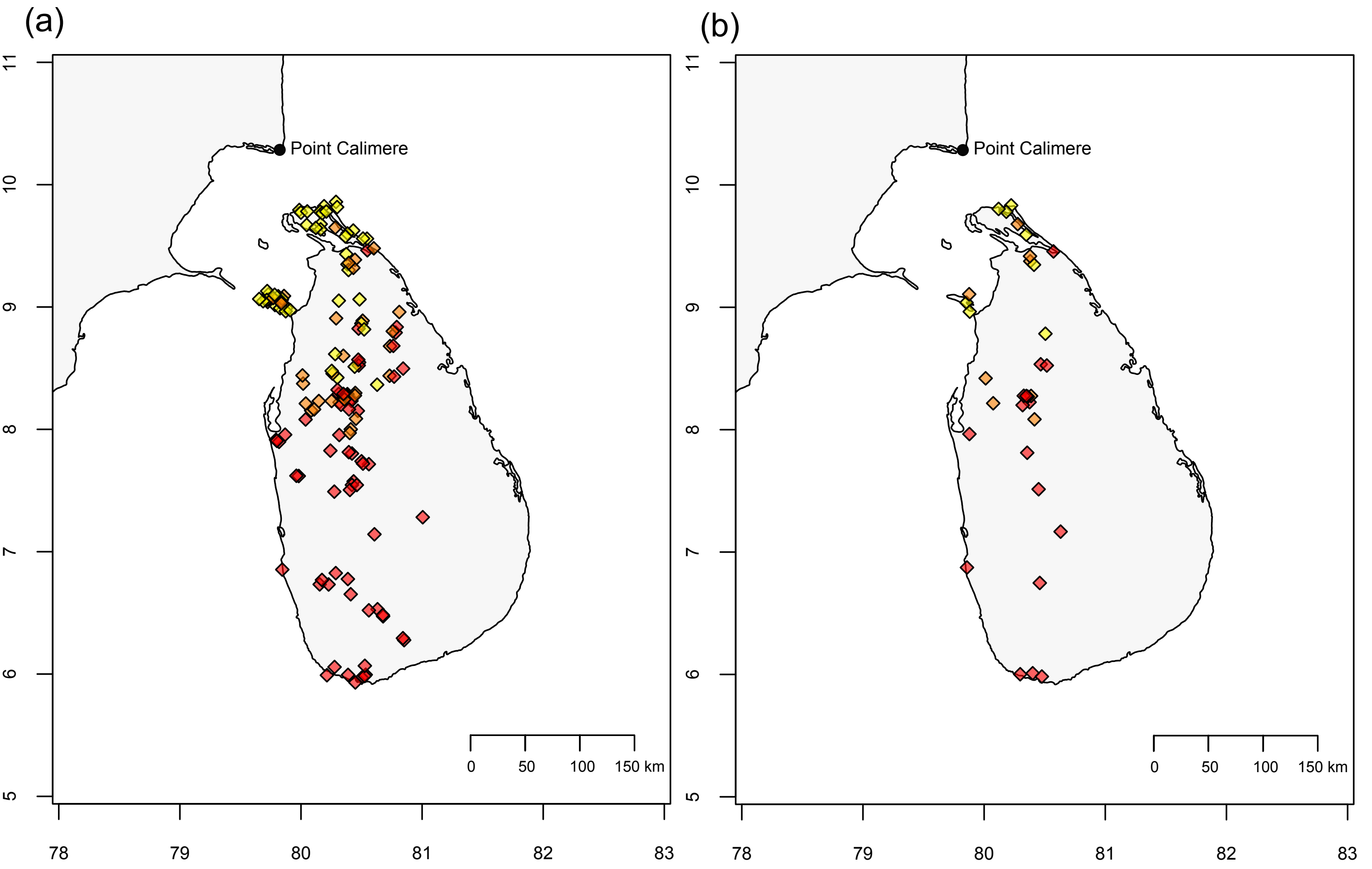

Figure S1: Sampling maps. (a) Map showing all sampled flamebacks (n = 141). (b) Map indicating samples excluded from downstream analysis due to high levels of missing genotype data (n = 33). The color of each point represents the phenotype of the bird: red for red-backed, yellow for yellow-backed, and orange for orange-backed individuals.

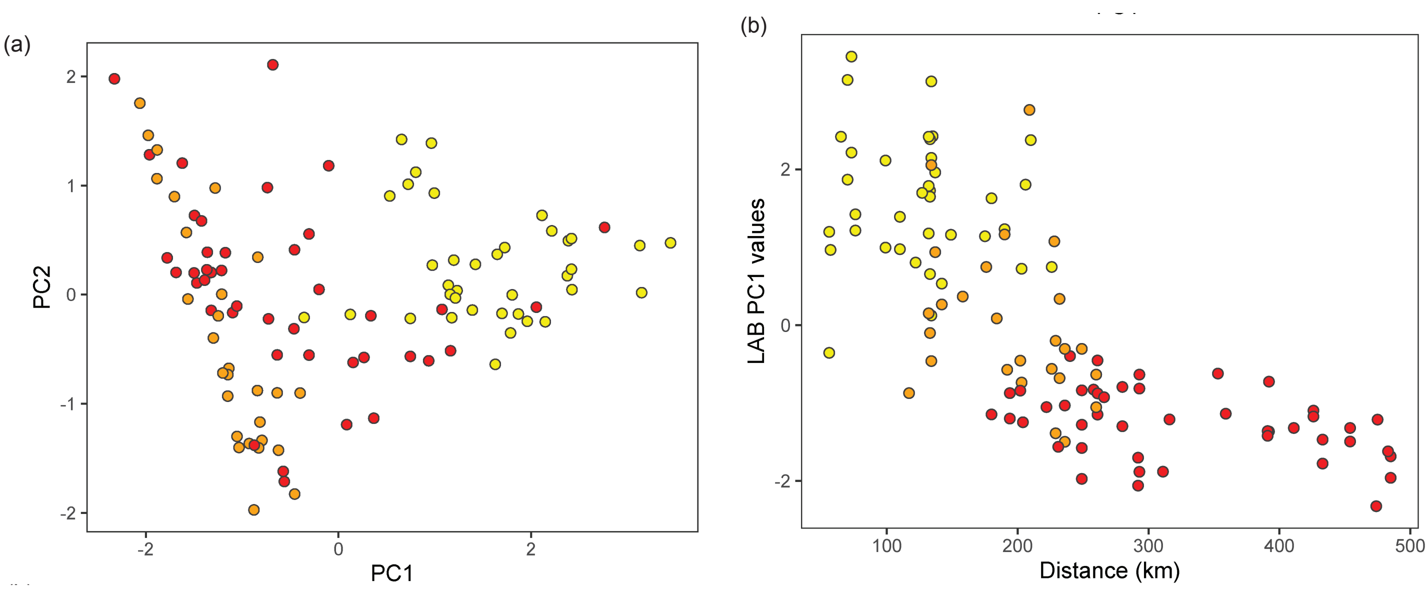

Figure S2: (A) Principal Component Analysis (PCA) based on L, a, b values. Principal Component (PC) 1 was utilized as the phenotypic score for the phenotypic cline analysis. Colors represent different species and hybrids: Red; *D. psarodes*, yellow; *D. benghalense*, and orange for hybrids. (B) PC 1 of the PCA based on L, a, b values plotted against geographical distance from Point Calimere to each sampling location, which was used for the cline analysis.
